# Significant Physical and Exercise-Related Variables for Exercise-Centred Lifestyle: Big Data Analysis for Gynaecological Cancer Patients

**DOI:** 10.1155/2021/5362406

**Published:** 2021-12-16

**Authors:** Eun Joo Yang, Hyunseok Jee

**Affiliations:** ^1^Department of Rehabilitation Medicine, Seoul National University Bundang Hospital, Gyeonggi-do, Republic of Korea; ^2^School of Kinesiology, Yeungnam University, 280 Daehak-ro, Gyeongsan, Gyeongbuk 38541, Republic of Korea

## Abstract

This study investigated the characteristics of gynaecological cancers and is aimed at identifying significant risk variables using the National Health Insurance Sharing Service database to develop practical interventions for affected patients. Data regarding patients with uterine and ovarian cancer from the National Health Insurance Sharing Service database were collected and analysed using Student's *t*-test, logistic regression, and receiver operating characteristic curve analyses. Student's *t*-test analyses revealed that age, body mass index, blood pressure, and waist variables differed significantly among patients with uterine cancer. Gamma-glutamyl transpeptidase levels were higher in patients with ovarian cancer than in patients with uterine cancer. Physical fitness function tests reflected the status of patients with cancer. Moreover, physical disability was associated with an increased incidence of ovarian cancer. Intensive exercise for 20 min more than 1 time per week must be avoided to prevent uterine cancer. Receiver operating characteristic curve analyses showed that the optimal cutoff value for one-leg standing time, a prognostic and preventive factor in ovarian cancer, was 9.50 s (sensitivity, 94.9%; specificity, 96.9%). Controlling significant variables for each gynaecological cancer type in an individualised and optimised manner is recommended, including by maintenance of an adjusted exercise-centred lifestyle.

## 1. Introduction

According to the 2016 gynaecological cancer-related report of Statistics Korea (http://kostat.go.kr/portal/eng/index.action), 44,367 and 3,538 individuals had uterine and ovarian cancers (6^th^ and 17^th^ most common cancers in Korea), respectively. Careful diagnosis should be performed for gynaecological cancers, which show tell-tale symptoms (i.e., postmenopausal bleeding or irregular bleeding) and is commonly diagnosed in the early stages. Gynaecological cancers have high recurrence rates and low survival rates. Moreover, specific aetiological factors contributing to gynaecological cancers are unclear, except for human papillomavirus infection, which causes cervical cancer [1]. Although the survival rate has increased due to medical treatments, including surgery, postoperative complications such as anaemia, malnutrition, and depression often occur [2, 3]. Nevertheless, there has been gradual progress in identifying modifiable variables that can severely affect the incidence of cancer, as well as other dependent factors that vary among cancer types [4]. Among these variables, physical activity considerably and positively improved both cancer symptoms and postoperative recovery. Prescribed exercise is adjusted based on the cancer target and involves considerations of critical disease-related factors [5]; however, specific prescribed exercise has not been well tailored to patients with gynaecological cancer.

Korean individuals are required to register their regular health check-up data, which enables the pooling of massive health-related datasets. The Korean government arranged electronic data updates beginning with the 2002 dataset pool, which is arranged within the National Health Insurance Sharing Service (NHISS) Database (DB) (https://www.nhis.or.kr) [6]. This DB allows researchers to perform prospective, retrospective, cross-sectional, and longitudinal studies. Studies using this massive DB have been performed to provide practical guidance for evidence-based interventions (e.g., exercise prescriptions) for target diseases [7].

This study aimed to determine the characteristics of gynaecological cancers (specifically uterine and ovarian cancer) and identify significant NHISS DB-derived risk variables among patients with these cancer types to provide beneficial information for the development of practical interventions. This study evaluated the following hypotheses:

1. There are significant gynaecological cancer-dependent risk variables that can be related to clinical benefits during targeted cancer treatment (i.e., risk factors for developing cancer or cancer prognosis)
2. Exercise prescription-orienting markers and physical function-related variables were identified using logistic regression analyses
3. By screening risk variables using logistic regression analyses, optimal threshold-related cutoff values can be obtained using receiver operating characteristic (ROC) curve analyses

## 2. Materials and Methods

### 2.1. The Data Source for Gynaecological Cancer Study and Its Demographic Characteristics

**Figure 1:**
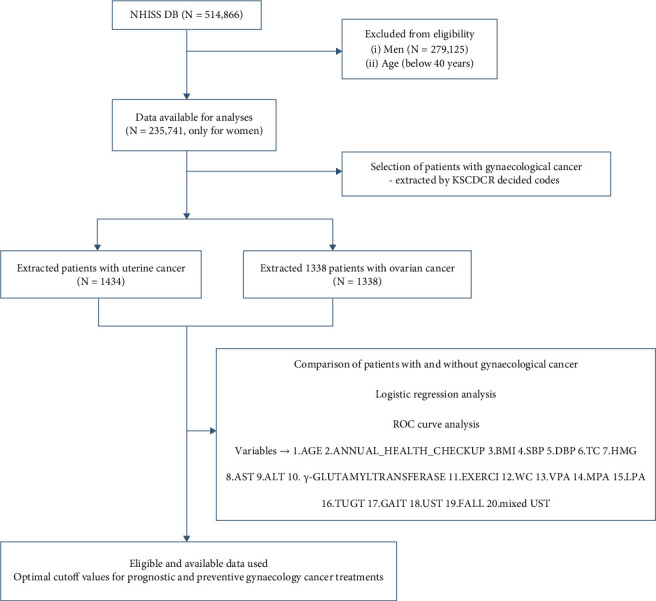
Study flowchart for selection and analyses of patients with gynaecological cancer. Data from 2002 to 2013 registered in the NHISS DB were used in this study. In total, 235,741 women were included in this study. KSCDCR-based uterine and ovarian cancer-related codes were used to extract the target cancer groups. As described in the Materials and Methods, Student's *t*-tests, logistic regression analyses, and ROC curve analyses were used to identify significant variables among patients with cancer and patients with benign lesions. NHISS DB: National Health Insurance Sharing Service Database; KSCDCR: Korean Standard Classification of Disease and Causes of Death; ROC: receiver operating characteristic.

All Korean-sourced medical records since 2002, collected during regular medical health check-ups, were gathered in electronic format in the NHISS DB (https://nhiss.nhis.or.kr/bd/ab/bdaba000eng.do). The NHISS DB used in the study included records of 514,866 randomly selected individuals aged >40 years. The DB included data regarding sex, age, medical insurance fee, resident region, and death, and all data were used for patient stratification. Of the 514,866 patients registered in the NHISS DB, only female patients (*n* = 235,741) were included in this study (Figure 1). The demographic and categorical characteristics of the patients included in this study are shown in Tables 1 and 2, respectively.

The study protocol was approved by the Institutional Review Board of the Seoul National University Bundang Hospital (X-1707-411-903). All participants were anonymously registered using their ID numbers to secure their personal information. The IRB determined that the NHISS data were exempt from the requirement for consent.

### 2.2. Study Design

Data of patients with uterine and ovarian cancers used in this cohort study were obtained from the NHISS-derived longitudinal DB. Using the Korean Standard Classification of Disease and Cause of Death (http://kssc.kostat.go.kr/ksscNew_web/index.jsp) to select codes for gynaecological cancers, 1,434 patients with uterine cancer, and 1,338 patients with ovarian cancer were extracted from this database. The following codes were used for uterine cancer: D06, carcinoma in situ of the cervix uteri; D06.0, carcinoma *in situ* of the endocervix; D06.1, carcinoma *in situ* of the exocervix; D06.7, carcinoma *in situ* of the other parts of the cervix; D06.9, carcinoma *in situ* of the cervix; and unspecified, and D07.0, carcinoma *in situ* of the endometrium. The following codes were used for ovarian cancer: C56, malignant neoplasm of the ovary; C56.0, malignant neoplasm of the ovary, right; C56.1, malignant neoplasm of the ovary, left; and C56.9, malignant neoplasm of the ovary, unspecified side. The codes for the control groups were as follows: D26, other benign neoplasms of the uterus and D27, benign neoplasm of the ovary. The variables necessary to analyse the detailed statuses of patients regarding carcinogenesis were selected and subsequently calculated using Student's *t*-tests, logistic regression analyses, and ROC curve analyses (Figure 1), while also considering benign diseases of each corresponding cancer group.

### 2.3. Variables Used in This Study

The following variables were used in this study: AGE, age (years); ANNUAL_HEALTH_CHECKUP, health check-up year (year); BMI, body mass index (kg/m^2^); SBP, systolic blood pressure (mmHg); DBP, diastolic blood pressure (mmHg); TC, total cholesterol (mg/dL); HMG, haemoglobin (g/dL); AST, serum glutamic oxaloacetic transaminase and aspartate aminotransferase (U/L); ALT, serum glutamic pyruvic transaminase and alanine aminotransferase (U/L); *γ*-glutamyl transpeptidase, gamma-glutamyl transpeptidase (U/L); EXERCI, frequency of moderate-intensity exercise per week (1, no; 2, 1–2 times; 3, 3–4 times; 4, 5–6 times; and 5, every day); WC, waist circumference (cm); VPA, frequency of high-intensity exercise > 20 min (1, 0 days/week; 2, 1 day/week; 3, 2 days/week; 4, 3 days/week; 5, 4 days/week; 6, 5 days/week; 7, 6 days/week; and 8, 7 days/week); MPA, frequency of moderate-intensity exercise > 30 min (1, 0 days/week; 2, 1 day/week; 3, 2 days/week; 4, 3 days/week; 5, 4 days/week; 6, 5 days/week; 7, 6 days/week; and 8, 7 days/week); LPA, frequency of light-intensity exercise/walking > 30 min (1, 0 days/week; 2, 1 day/week; 3, 2 days/week; 4, 3 days/week; 5, 4 days/week; 6, 5 days/week; 7, 6 days/week; and 8, 7 days/week); timed up and go test (TUGT), time in seconds for standing up from a chair, walking 3 m, and returning to the same chair to measure basic mobility; GAIT, gait disability (1, yes and 2, no); unipedal stance test (UST), standing time on one leg in seconds to assess static postural and balance control for monitoring neurological and musculoskeletal status, and for managing fall risk; mixed UST, standing on one leg (1, with eyes closed and 2, with eyes open); and FALL, experiencing a fall within 6 months (1, yes and 2, no).

### 2.4. Statistical Analyses

All data are presented as the mean ± standard deviation. Student's *t*-tests and logistic regression analyses were used to evaluate differences between the gynaecological cancer and noncancer groups. The *p* values were not adjusted for multiple comparisons. ROC curve analyses were used to determine the optimal cutoff values for the significant variables identified via logistic regression analyses. SAS software (version 9.4; SAS Institute, Cary, NC, USA) was used for all statistical analyses. Statistical significance was set at *p* < 0.05.

## 3. Results

### 3.1. Characteristics of Gynaecological Cancer and Nongynaecological Cancer Groups

Age, BMI, systolic and diastolic blood pressures, and waist circumference were significantly different between patients with uterine cancer and the control group (*p* < 0.01). In contrast, gamma (*γ*)-glutamyl transpeptidase levels were significantly higher in patients with ovarian cancer than in patients with benign ovarian lesions (*p* < 0.01). Significant differences in physical function-related variables (e.g., unipedal stance test and timed up and go test) (*p* < 0.05) were observed among patients with cancer (Table 3).

### 3.2. Logistic Regression Analyses of Nonphysical Activity-Related Variables

Logistic regression analyses showed cancer type-specific trends for age, BMI, serum glutamic oxaloacetic transaminase and aspartate aminotransferase, serum glutamic pyruvic transaminase and alanine aminotransferase, *γ*-glutamyl transpeptidase, and waist circumference (*p* < 0.05).

Increased times in the timed up and go test and unipedal stance test were associated with a greater incidence of ovarian cancer (*p* < 0.05). Significant differences in mixed unipedal stance test results were observed in patients with ovarian cancer (odds ratio (OR), 0.55; 95% confidence interval (CI), 0.400–0.78; *p* < 0.01). Moreover, a significant difference was observed between patients with ovarian cancer and those with uterine cancer in terms of experiencing falls within 6 months (*p* < 0.01). Patients with no falls during the preceding 6 months had a lower incidence of ovarian cancer (OR, 0.70) (Table 3).

### 3.3. Results of Logistic Regression Analyses on Exercise-Dependent Modalities

Patients were asked about various exercise modalities with different intensities, durations, and frequencies (Table 4). Numbers next to the variables show a significant difference in the selected questionnaire. For example, the frequency of moderate-intensity exercise per week (1) [4] indicates that 1 and 4 patients with uterine and ovarian cancers, respectively, responded to the questionnaire. Regarding the frequency of high-intensity exercise > 20 min in patients with uterine cancer, engagement in this exercise for 1 d per week demonstrated significant differences (OR, 1.55; 95% CI, 1.14–2.12; *p* < 0.01). Regarding moderate-intensity exercise for >30 min in patients with uterine cancer, engagement in this exercise for 1 d per week demonstrated significant differences (OR, 1.76; 95% CI, 1.30–2.37; *p* < 0.01). In patients with ovarian cancer, the frequency of 5 days/week engagement in light-intensity exercise/walking > 30 min was the only significant difference (OR, 1.61; 95% CI, 1.08-2.39).

### 3.4. ROC Curve Analyses of Gynaecological Cancer

**Figure 2:**
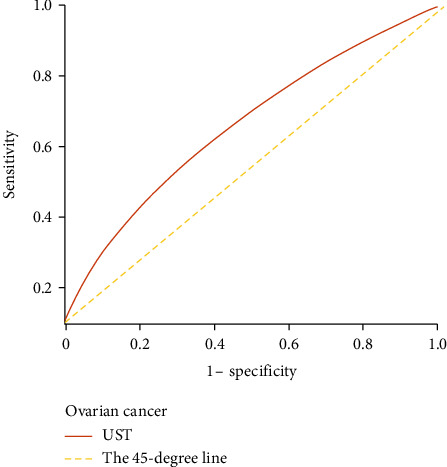
Depiction of ROC curve analysis. ROC curve analyses of UST (unipedal stance test) results showed an optimal threshold value of 9.5 s for patients with ovarian cancer (*p* < 0.05; sensitivity, 94.9%; and specificity, 96.9%; Table 5). The 45-degree line is shown to facilitate comparison with the UST line. ROC: receiver operating characteristic; UST: unipedal stance test (standing time on one leg in seconds to assess static postural and balance control for monitoring neurological and musculoskeletal status, and for managing fall risk).

For ovarian cancer, the cutoff value of *γ*-glutamyl transpeptidase was 14.50 (U/L, with a sensitivity of 70.7% and specificity of 71%. The UST results showed an optimal cutoff value of 9.50 s, with a sensitivity of 94.9% and specificity of 96.9% (*p* < 0.05) (Table 5). The ROC curve analysis of the UST results for ovarian cancer is shown in Figure 2.

## 4. Discussion

In this study, 1,434 patients with uterine cancer and 1,338 patients with ovarian cancer from the 514,866 registered NHISS DB retrospective cohort were enrolled [8]. The regression model in this study was originally used to compare patients with and without a target cancer. Significant differences among the variables associated with cancer incidence were identified between uterine and ovarian cancers. Specifically, patients with ovarian cancer had higher levels of *γ*-glutamyl transpeptidase (*p* < 0.001). Moreover, physical fitness functional test results reflected the inferior status of patients with cancer (*p* < 0.05), compared with patients who did not have cancer. Physical disabilities increased the incidence of ovarian cancer (OR, 1.01; *p* < 0.05). In contrast, patients with no falls during the preceding 6 months had a lower incidence of ovarian cancer (OR, 0.70). Furthermore, a 20 min intensive exercise session more than 1 time per week was associated with an increased incidence of uterine cancer (*p* < 0.05). ROC curve analysis showed that the optimal cutoff value for one-leg standing time, a prognostic and preventive factor in ovarian cancer, was 9.50 s (sensitivity, 94.9%; specificity, 96.9%). These findings can aid in the design of preventive and care exercise interventions for gynaecological cancer.

### 4.1. Patterns Observed from the Obtained Results

The measurement of continuous variables revealed two patterns (Table 3). Age in uterine cancer and *γ*-glutamyl transpeptidase in ovarian cancer were identified as significant influencing factors in logistic regression analyses (*p* < 0.05 and *p* < 0.01, respectively). Interestingly, *γ*-glutamyl transpeptidase levels were elevated in ovarian cancer patients who may be exposed to chemotherapy agents that affect liver function in the long term. Another possibility is that active cancer in the liver showed increased levels of *γ*-glutamyl transpeptidase. For age, ROC curve analyses showed a significant cutoff value of 41.5 years for patients with uterine cancer. The other variables did not exhibit significant differences, as shown in Table 3. Logistic regression analyses in an important previous study showed that diastolic blood pressure and frequency of moderate-intensity exercise per week 2 (1–2 times per week) were significant influencing factors in patients with colorectal cancer [9]. In general, obesity-related variables were found to be significant influencing factors. In our clinical experience, endometrioma markedly differs from ovarian cancer in terms of physical function, because patients with endometrioma tend to have obesity and decreased physical activity.

### 4.2. Obesity-Related Variables Identified in Previous Studies

The current study findings were consistent with a previous report regarding cancer levels classified according to the effects of obesity. In patients with cancer, leukaemia, pancreatic carcinoma, uterine carcinoma, and colorectal carcinoma contribute to gynaecological cancer, and obesity (BMI) is the presenting sign [10]. The results reported by McTiernan et al. were consistent with the findings of the current study, such that overweight and obesity statuses were associated with the development of gynaecological cancer and elevated patient mortality [11]. Among patients with ovarian cancer, 30% were overweight, and 12% were obese [12]. According to an aetiological study at the molecular level, elevated levels of inflammatory factors (e.g., tumour necrosis factor-alpha, interleukins 1 and 6, and C-reactive protein levels) were observed in patients with cancer and were linked to reduced survival [13–15]. Although patients can survive cancer that is aggravated by severe obesity, they continue to experience obesity-related comorbidities such as hypertension, diabetes, osteoarthritis, and cardiopathy after cancer treatment [16, 17]. Patients who are overweight and obese are also likely to develop physical disabilities, which may affect their risk status.

### 4.3. Effects of Exercise-Related Interventions on Cancer Status

Concerning the effect of exercise on cancer status, few studies have investigated the appropriate intensity of exercise intervention for patients with cancer. The results of this study suggest that the modalities (e.g., intensity, duration, and frequency) of exercise intervention should depend on the cancer type. Notably, among patients with uterine cancer, engagement in high-intensity exercise for >20 min for 1 d per week had an OR of 1.55. Hence, 20 min of high-intensity exercise more than 1 time per week must be avoided to prevent uterine cancer. Overall, the findings suggest that >2 days of 30 min moderate-intensity exercise may be ideal for patients with uterine cancer (Table 4).

Furthermore, the UST could be a more appropriate measure for patients with ovarian cancer than for patients with uterine cancer (*p* < 0.05) (Tables 3 and 5). Falls in the preceding 6 months were much less common in patients with ovarian cancer (OR, 0.70; 95% CI, 0.55–0.89) than in patients with uterine cancer (OR, 1.49; 95% CI, 1.12–1.98; *p* < 0.01). This may be attributable to many factors such as age, carcinogenesis causes, disease symptoms, and detailed body locations and functions, although the diseases are both classified as gynaecological cancers. The results of the physical function-related analyses were consistent in patients with ovarian cancer (Table 5). Moreover, the lack of physical capacity, as a cancer symptom, might increase the risk of falls, although this depends on the type of cancer.

In our clinical experience, patients with ovarian cancer receive comparatively more chemotherapy than other patients with cancer, contributing to reduced sensations in the feet. This is consistent with changes in walking-related variables, such as the TUGT and UST results. Patients with gynaecological cancer are trapped in a vicious cycle, whereby decreased sensation of balance during walking is caused by neuromuscular impairment, which then induces core muscle weakening, resulting in less participation in rehabilitation programs. Moreover, studies in the past decade have shown that the socioeconomic cost of injury due to falls is approximately 343,000,000,000 Korean won, and 32% of older patients experience falls [18–20].

Finally, ROC curve analyses (Table 5) showed that UST results had a significant optimal cutoff value of 9.5 s (sensitivity, 94.9%; specificity, 96.9%), which can be used to design exercise intervention programs for patients with ovarian cancer.

The limitations of this study include its cross-sectional design, which led to a greater emphasis on associations between significant variables (e.g., exercise duration and type) and cancer symptoms (carcinogenetic repression), rather than on aetiological understanding. However, aetiological associations can be identified using logistic regression analyses [9], as in this study. The significant findings from the regression models used in this study should be considered for practical applications.

## 5. Conclusions

The following novel findings were obtained from the NHISS DB analysis of patients with gynaecological cancer:

1. Physical disabilities increased the incidence of ovarian cancer (OR, 1.01; *p* < 0.05). Patients with no falls during the preceding 6 months had a lower incidence of ovarian cancer (OR, 0.70). Exmination of four exercise modalities showed that a 20 min intensive exercise session (1 time per week) was associated with an increased incidence of uterine cancer (*p* < 0.01)
2. ROC curve analyses showed that the optimal cutoff value for one-leg standing time, a prognostic and preventive factor in ovarian cancer, was 9.50 s (sensitivity, 94.9%; specificity, 96.9%)

Moreover, significant variables (e.g., age, waist circumference, BMI, and hepatic- and blood pressure-related variables) varied according to cancer type. Similarly, some variables exhibited a pattern such as those related to physical fitness function. Therefore, exercise interventions must be tailored to target cancer symptoms, with careful consideration of the significant variables identified in this large-scale NHISS DB study.

## Figures and Tables

**Table 1:** Demographic characteristics of patients with gynaecological cancer from NHISS DB.

Variables	Uterine		Ovarian	
	Cancer	Non-cancer	Cancer	Non-cancer
Age (y)	44.4 ± 3.4	54.2 ± 9.5	50.9 ± 9.0	51.1 ± 9.1
BMI (kg/m^2^)	23.4 ± 2.9	24.1 ± 3.2	24.0 ± 3.3	23.9 ± 3.0
SBP (mmHg)	119.1 ± 13.6	125.6 ± 16.4	123.7 ± 15.9	123.7 ± 16.0
DBP (mmHg)	74.5 ± 9.2	76.4 ± 10.4	76.0 ± 10.0	76.0 ± 9.9
TC (mg/dL)	202.5 ± 37.1	202.3 ± 38.1	205.0 ± 38.0	205.3 ± 37.7
AST	25.8 ± 21.5	25.3 ± 13.2	26.3 ± 32.4	25.4 ± 11.7
ALT	23.5 ± 27.6	22.1 ± 16.1	22.6 ± 16.8	22.0 ± 12.5
*γ*-Glutamyl transferase (U/L)	25.4 ± 28.6	23.8 ± 25.7	27.6 ± 51.9	23.7 ± 21.3
WC (cm)	76.9 ± 7.9	79.8 ± 8.3	78.9 ± 8.4	79.0 ± 8.2
TUGT (s)	8.1 ± 1.2	8.2 ± 1.4	8.4 ± 1.5	8.3 ± 1.4
UST (s)	13.7 ± 11.4	14.2 ± 11.0	15.6 ± 13.2	13.9 ± 11.1

Values are presented as mean ± standard deviation. Demographic characteristics of continuous variables in patients with gynaecological cancer are shown. NHISS DB: National Health Insurance Sharing Service Database; SBP: systolic blood pressure; DBP: diastolic blood pressure; TC: total cholesterol; AST: serum glutamic oxaloacetic transaminase and aspartate aminotransferase; ALT: serum glutamic pyruvic transaminase and alanine aminotransferase; WC: waist circumference; TUGT: timed up and go test (time in seconds for standing up from a chair, walking 3 m, and returning to the same chair to measure basic mobility); UST: unipedal stance test (standing time on one leg in seconds to assess static postural and balance control for monitoring neurological and musculoskeletal status, and for managing fall risk).

**Table 2:** Categorical characteristics of patients with gynaecological cancer.

Variables		Uterine (%)		Ovarian (%)	
		Cancer	Noncancer	Cancer	Noncancer
Annual health check-up	2002–2007	89 (10.8)	423 (18.1)	298 (22.3)	209 (13.2)
	2008–2013	739 (89.3)	1919 (81.9)	1040 (77.7)	1378 (86.8)

VPA	1	458 (62.7)	1308 (69.0)	673 (66.7)	896 (66.0)
	2	138 (18.9)	277 (14.6)	163 (16.2)	230 (17.0)
	3	80 (11.0)	171 (9.0)	106 (10.5)	142 (10.5)
	4	40 (5.5)	88 (4.6)	41 (4.1)	68 (5.0)
	5	14 (1.9)	52 (2.7)	26 (2.6)	21 (1.6)

MPA	1	403 (55.1)	1180 (62.2)	566 (56.1)	788 (58.1)
	2	146 (20.0)	284 (15.0)	206 (20.4)	238 (17.5)
	3	113 (15.5)	238 (12.6)	138 (13.7)	193 (14.2)
	4	49 (6.7)	108 (5.7)	60 (6.0)	84 (6.2)
	5	20 (2.7)	87 (4.6)	39 (3.9)	54 (4.0)

LPA	1	235 (32.2)	610 (32.1)	302 (30.0)	430 (31.7)
	2	137 (18.8)	292 (15.4)	188 (18.7)	244 (18.0)
	3	152 (20.8)	407 (21.4)	224 (22.2)	285 (21.0)
	4	111 (15.2)	285 (15.0)	155 (15.4)	196 (14.5)
	5	95 (13.0)	304 (16.0)	139 (13.8)	201 (14.8)

Fall	1	66 (8.9)	247 (12.6)	162 (15.2)	156 (11.1)
	2	679 (91.1)	1708 (87.4)	907 (84.9)	1247 (88.9)

Mixed UST	1	23 (3.1)	83 (4.3)	84 (8.1)	64 (4.6)
	2	717 (96.9)	1834 (95.7)	956 (91.9)	1314 (95.4)

Values in parentheses are percentages (%). LPA: light physical activity/walking > 30 min; MPA: moderate physical activity > 30 min; VPA: vigorous physical activity > 20 min (1, 0 days; 2, 1–2 days; 3, 3–4 days; 4, 5–6 days; and 5, 7 days); Fall: experiencing a fall within 6 months (1, yes and 2, no); mixed UST: unipedal stance test (1, with eye open and 2, closed).

**Table 3:** Logistic regression analyses of variables affecting gynaecological carcinogenesis.

	Uterine cancer			Ovarian cancer		
	*p* value	OR	95% CI	*p* value	OR	95% CI
AGE	*p* ≤ 0.001^∗∗^	0.81	0.80-0.83	0.096	0.99	0.978-1.002^#^
BMI	0.085	0.95	0.90-1.01	0.759	1.01	0.96-1.05
SBP	0.083	0.99	0.98-1.00	0.631	1.00	0.99-1.01
DBP	0.123	1.01	1.00-1.03	0.686	1.00	0.985-1.010^#^
TC	0.501	1.00	0.998-1.004^#^	0.445	1.00	0.997-1.001^#^
AST	0.400	1.01	0.99-1.02	0.154	0.99	0.98-1.00
ALT	0.563	1.00	0.998-1.006^#^	0.695	1.00	0.99-1.01
*γ*-Glutamyl transpeptidase	0.311	1.00	0.998-1.005^#^	0.019∗	1.005#	1.001-1.008^#^
WC	0.719	1.00	0.98-1.03	0.703	1.00	0.98-1.01
TUGT	0.040^∗^	0.93	0.88-1.00	0.060	1.06	1.00-1.12
UST	0.350	1.00	0.988-1.044^#^	0.002^∗∗^	1.01	1.00-1.02
FALL	0.006^∗∗^	1.49	1.12-1.98	0.003^∗∗^	0.70	0.55-0.89
Mixed UST	0.151	1.41	0.88-2.26	0.001^∗∗^	0.55	0.400-0.78

The OR values of each variable are shown according to patients with and without uterine or ovarian cancer. ^∗^*p* < 0.05, ^∗∗^*p* < 0.01: statistically significant differences; variables with significant *p* values were selected via logistic regression analyses. ^#^Three decimal places as rounding OR and 95% CI off to the nearest thousandth is not desirable (for example, 95% CI, 1.00–1.00). OR: odds ratio; CI: confidence interval; AGE: age (years); BMI: body mass index (kg/m^2^); SBP: systolic blood pressure (mmHg); DBP: diastolic blood pressure (mmHg); TC: total cholesterol (mg/dL); AST: serum glutamic oxaloacetic transaminase and aspartate aminotransferase (U/L); ALT: serum glutamic pyruvic transaminase and alanine aminotransferase (U/L); *γ*-glutamyl transpeptidase (U/L); WC: waist circumference (cm); TUGT: timed up and go test, time in seconds for standing up from a chair, walking 3 m, and returning to the same chair; UST: unipedal stance test (standing time on one leg in seconds to assess static postural and balance control for monitoring neurological and musculoskeletal status, and for managing fall risk); FALL: experiencing a fall within 6 months (1, yes and 2, no); mixed UST: standing on one leg (1, with eyes closed and 2, with eyes open).

**Table 4:** Multivariable relative risk of gynaecological cancer by activity subtype.

	Uterine cancer			Ovarian cancer		
	Duration (days/week)	OR (95% CI)	*p* value	Duration (days/week)	OR (95% CI)	*p* value
LPA	0	1.37 (0.98–1.93)	0.067	5	1.61 (1.08–2.39)	0.018^∗^
MPA	1	1.76 (1.30–2.37)	<0.001^∗∗^	0	1.21 (0.91–1.60)	0.190
VPA	1	1.55 (1.14–2.12)	0.006^∗∗^	6	1.65 (0.92–2.96)	0.093

The selected number in the duration column indicates the variable with the most significant *p* value; for example, the number “0” (no exercise days during the week) was selected for the LPA of uterine cancer because it had the lowest *p* value (0.067), compared with other numbers of exercise days during the week. ^∗^*p* < 0.05, ^∗∗^*p* < 0.01: statistically significant; OR: odds ratio; CI: confidence interval; LPAL: light physical activity (walking) > 30 min; MPA: moderate physical activity > 30 min; VPA: vigorous physical activity > 20 min.

**Table 5:** ROC curve analyses of patients with gynaecological cancer.

Cancer	Variable	AUC	*p* value	Sensitivity (%)	Specificity (%)
Ovarian cancer	*γ*-Glutamyl transpeptidase	0.511	0.361	70.7	71.0
	UST	0.524	0.040^∗^	94.9	96.9

Variables with significant *p* values were extracted by logistic regression analyses, and optimal cutoff points were determined via ROC curve analyses. Cutoff values were those with a concomitant sensitivity and specificity of >70%. ^∗^*p* < 0.05: statistically significant differences; ROC: receiver operating characteristic; AUC: area under the curve; UST: unipedal stance test (standing time on one leg in seconds to assess static postural and balance control for monitoring neurological and musculoskeletal status, and for managing fall risk); *γ*-glutamyl transpeptidase (U/L).

## Data Availability

Publicly available datasets were analysed in this study. These data can be found at https://nhiss.nhis.or.kr.
